# Local immunity by tissue-resident CD8^+^ memory T cells

**DOI:** 10.3389/fimmu.2012.00340

**Published:** 2012-11-09

**Authors:** Thomas Gebhardt, Laura K. Mackay

**Affiliations:** Department of Microbiology and Immunology, The University of MelbourneMelbourne, VIC, Australia

**Keywords:** T cell, memory, protection, periphery, virus infection

## Abstract

Microbial infection primes a CD8^+^ cytotoxic T cell response that gives rise to a long-lived population of circulating memory cells able to provide protection against systemic reinfection. Despite this, effective CD8^+^ T cell surveillance of barrier tissues such as skin and mucosa typically wanes with time, resulting in limited T cell-mediated protection in these peripheral tissues. However, recent evidence suggests that a specialized subset of CD103^+^ memory T cells can permanently lodge and persist in peripheral tissues, and that these cells can compensate for the loss of peripheral immune surveillance by circulating memory T cells. Here, we review evolving concepts regarding the generation and long-term persistence of these tissue-resident memory T cells (T_RM_) in epithelial and neuronal tissues. We further discuss the role of T_RM_ cells in local infection control and their contribution to localized immune phenomena, in both mice and humans.

## Introduction

Primary infection results in immunity against reencounter with the same pathogen. This anamnestic “immune memory” builds the conceptual basis for vaccination and relies on different types of adaptive memory cells including B cells and T cells (Welsh et al., 2004). While almost all current vaccines target B cells for the induction of antibody-mediated immunity, this approach has significant limitations for a number of infectious diseases including AIDS, tuberculosis, and malaria (Seder and Hill, 2000). The relative inefficiency of humoral immunity in these cases is related to various evasion strategies employed by the pathogens, including their rapid variation of exposed antigens and their intracellular localization. In addition, serum antibodies have only limited access to some epithelial compartments under non-inflammatory conditions and as a consequence, may fail to provide the heightened level of immediate immune control required to prevent the establishment and persistence of peripheral infections (Zinkernagel, 2002). Thus, protection from certain pathogens relies on potent T cell-dependent cellular immune responses (Seder and Hill, 2000). Understanding the generation and maintenance of such T cell responses, particularly those in barrier locations at body surfaces, is of critical importance for the development of future vaccines.

## Circulating CD8^+^ memory T cells

The activation of pathogen-specific T cells in lymphoid tissues draining the site of infection results in the generation of a large pool of effector cells (Jameson and Masopust, 2009). During activation, these cells acquire the expression of certain migration receptors that allow them to infiltrate peripheral tissues where they contribute to pathogen clearance through the elimination of infected cells and the release of proinflammatory and microbicidal mediators (Harty et al., 2000; Nolz et al., 2011). Interestingly, the organ-specific microenvironments in lymphoid tissues can preferentially support the induction of migration receptors required for T cell entry specifically into those tissues that are connected to the respective lymphoid priming sites (Agace, 2006; Nolz et al., 2011). Such organ-specific migration imprinting is best documented for barrier tissues such as skin and gut and involves the induction of ligands for E- and P-selectin as well as the integrin α 4β 7 and the chemokine receptor CCR9, respectively (Parrott et al., 1976; Guy-Grand et al., 1978; Campbell and Butcher, 2002; Mora et al., 2005; Agace, 2006; Nolz et al., 2011). It should be noted, however, that migration imprinting is by no means absolute and that substantial numbers of effector CD8^+^ T cells disperse into a broad variety of peripheral tissues even after localized peripheral infections (Marshall et al., 2001; Masopust et al., 2004).

While the vast majority of effector cells disappear from the circulation following pathogen clearance, there remains a heterogeneous population of long-lived memory cells capable of mounting rapid recall responses on antigen reencounter (Jameson and Masopust, 2009). Memory T cells are commonly grouped into two major subsets based on their functional status and expression of certain homing receptors: (1) central memory T cells (T_CM_) that express the lymph node-targeting molecules CD62L and CCR7, and (2) effector memory T cells (T_EM_) that largely lack these receptors, but instead express certain migration molecules that target them to peripheral non-lymphoid tissues (Sallusto et al., 1999, 2004). This delineation between the two subsets, as initially defined for human blood cells (Sallusto et al., 1999), is now also widely used to define migratory abilities and the anatomical localization of memory subsets in other species (Seder and Ahmed, 2003).

Early migration studies employing adoptive transfer and lymph canulation experiments in sheep, described memory T cells as constantly recirculating cells that patrol through peripheral tissues including the skin and intestinal mucosa (Gowans and Knight, 1964; Cahill et al., 1977; Mackay et al., 1990, 1992). These seminal studies along with more recent work using parabiotic systems in mice, have led to the concept that blood-borne memory cells frequently exchange with their counterparts in lymphoid and most extra-lymphoid or peripheral tissues (Butcher and Picker, 1996; Von Andrian and Mackay, 2000), although exceptions for some epithelial and neuronal tissues with limited exchange between blood and tissue cells have been noted (Klonowski et al., 2004). Consistent with the subset-specific expression of migration receptors, peripheral tissues almost exclusively contain CD62L^−^ cells which, at face value, appear to fit into the T_EM_ category (Masopust et al., 2004). Lymph nodes however, harbor both CD62L^+^ and CD62L^−^ memory cells, albeit showing a strong bias toward T_CM_ cells (Sprent and Surh, 2002) that are likely to have entered the lymph nodes from the blood by extravasating through high endothelial venules in a CD62L- and CCR7-dependent fashion (Gallatin et al., 1983; Warnock et al., 1998; Forster et al., 1999). The minor fraction of CD62L^−^ cells found in lymph nodes instead may be comprised of classical T_EM_ cells that return from peripheral tissues via afferent lymphatics (Mackay et al., 1990, 1992).

Interestingly, while CCR7 is critically involved in T cell homing to lymph nodes via high endothelial venules (Forster et al., 1999), its expression is also required for efficient T cell entry into afferent lymphatic vessels in peripheral tissues (Bromley et al., 2005; Debes et al., 2005). This raises the important question as to whether peripheral T_EM_ cells dynamically regulate CCR7 expression during tissue exit and/or whether there may exist distinct populations of peripheral T cells with varying abilities to return to the circulation depending on their expression levels of CCR7 and their responsiveness to locally produced chemokines (Bromley et al., 2005). Regardless, it appears that the delineation of T cell memory into T_CM_ and T_EM_ subsets based on CD62L and CCR7 expression, may not reflect the full complexity of chemokine receptor usage in T cell recirculation that has now emerged from animal studies. Furthermore, recently described populations of non-migratory memory T cells in peripheral tissues that exist in disequilibrium with the circulating T cell pool, do not easily fit this widely used classification of T cell memory subsets.

## Immune protection by circulating CD8^+^ memory T cells

Memory T cells differ from their naïve counterparts by their persistence at elevated frequencies, their broader anatomical distribution and their ability to rapidly acquire effector functions upon reactivation (Kaech et al., 2002). As a consequence, they are regarded as powerful mediators of immune protection from reinfection. In keeping with this, protective immunity by CD8^+^ memory T cells has been demonstrated in various experimental models of systemic infection, such as *Listeria monocytogenes*, lymphocytic choriomeningitis virus (LCMV), and the malaria parasite *Plasmodium berghei* (Bachmann et al., 1997, 2005; Wherry et al., 2003; Badovinac et al., 2005; Huster et al., 2006; Schmidt et al., 2008). Importantly, T cell immunity in these cases of systemic infection is long-lived and can be conferred to naïve animals by the adoptive transfer of pathogen-specific CD8^+^ memory T cells (Lau et al., 1994). Thus, it appears that persisting antigen is not required for the maintenance of functional CD8^+^ T cell memory that is able to control lymphoid replication and systemic dissemination of various pathogens.

### Memory T cell migration and peripheral immune surveillance

In contrast to their role in immune protection from systemic infections, the ability of circulating CD8^+^ memory T cells to deal with localized infections in the periphery is surprisingly limited (Bachmann et al., 1997, 2005; Jiang et al., 2012; Mackay et al., 2012a). This lack of peripheral protection by circulating memory T cells, seen across a variety of models, is best explained by their progressive loss of peripheral migration abilities (Kundig et al., 1996; Bachmann et al., 1997; Woodland and Kohlmeier, 2009). Consistent with this, effector or early memory T cells in the circulation gradually lose expression of homing molecules required to enter peripheral tissues (Masopust et al., 2010; Gebhardt et al., 2011). In addition, the population of circulating T_EM_ cells appears to decline over time, resulting in a progressive conversion of the memory cell pool toward a CD62L^+^ T_CM_ phenotype (Tripp et al., 1995; Wherry et al., 2003). Collectively, these changes result in a skewing of the overall pattern of memory T cell recirculation from peripheral, non-lymphoid organs early after infection toward secondary lymphoid organs in the absence of continued antigenic stimulation (Gebhardt et al., 2011; Yang et al., 2011). Interestingly, this shift in migratory preference is most pronounced in the CD8^+^ T cell subset, whereas CD4^+^ T cells appear to retain their ability to migrate through peripheral tissues including the skin and intestinal mucosa, for prolonged periods of time (Gebhardt et al., 2011; Yang et al., 2011). In line with this, CD4^+^ memory T cells predominate among the population of peripheral cells returning to the circulation via afferent lymphatics in both sheep and humans (Mackay et al., 1990; Yawalkar et al., 2003). Taken together, it appears that continuous antigenic stimulation is essential for optimal migratory immune surveillance by CD8^+^ memory T cells, but not for their CD4^+^ counterparts. This requirement obviously poses significant challenges for the development of future vaccines targeting CD8^+^ T cell memory. Along these lines, recent studies have shown that ongoing T cell activation by persisting vaccine vectors is necessary for long-lived mucosal immunity against SIV infection in non-human primates (Hansen et al., 2009, 2011), highlighting the requirement for persisting antigenic stimulation to sustain ongoing peripheral CD8^+^ T cell surveillance.

## Permanently tissue-resident CD8^+^ memory T (T_RM_) cells

Despite the progressive loss of circulating T_EM_ cells with peripheral migration abilities, extra-lymphoid tissues including the intestinal and vaginal mucosa, skin, brain, salivary glands, and several others can harbor substantial populations of long-lived pathogen-specific CD8^+^ memory T cells (Table 1) (Bevan, 2011; Sheridan and Lefrancois, 2011; Ariotti et al., 2012). Of note, such cells are often localized to epithelial or neuronal tissues, meaning that their access to lymphatic vessels, located in the respective non-epithelial or non-neuronal compartments, is restricted by structural barriers such as basement membranes and the blood brain barrier, respectively. A common feature of CD8^+^ memory T cells in epithelial and neuronal tissues is their expression of high levels of CD103, the α-chain of the α_E_β_7_ integrin, CD49a, the α-chain of the α_1_β_1_ integrin [very late antigen (VLA-1)], and CD69, a surface molecule typically found on recently activated T cells. Interestingly, dendritic epidermal γδ T cells (DETC) in mouse skin, as well as other types of non-conventional T cells in epithelial tissues also express high levels of CD103 and CD69 (Ibraghimov and Lynch, 1994; Lefrancois et al., 1994; Jameson et al., 2004). This suggests that microenvironmental factors in these niches may be responsible for the sustained expression of high levels of these molecules on T cells. While naïve CD8^+^ T cells express intermediate levels of CD103, effector and resting memory T cells in the circulation lack expression of both CD103 and CD69 (Lefrancois et al., 1994; Masopust et al., 2006; Gebhardt et al., 2009). This noticeable phenotypic difference led to the hypothesis that circulating and peripheral memory T cells may represent distinct populations that show minimal, if any, intermixing with each other (Ibraghimov and Lynch, 1994; Masopust and Lefrancois, 2003). As discussed below, there is now compelling evidence that peripheral CD8^+^ CD103^+^ memory T cells indeed represent a non-migratory population of cells that are maintained without replenishment from the circulating memory T cell pool. Emphasizing these unique features, these cells are now widely referred to as tissue-resident memory T (T_RM_) cells (Bevan, 2011; Di Meglio et al., 2011; Sheridan and Lefrancois, 2011; Ariotti et al., 2012).

**Table 1:** **Examples of mouse models describing peripheral CD8^+^ memory T cells that fit the recent definition of CD103^+^ T_RM_ cells**.

**Category**	**Mode of lodgment**	**Location of T_RM_ cells**	**References**
Virus infection	Influenza virus infection (i.c.)	Brain	Hawke et al., 1998
	Lymphocytic choriomeningitis virus infection	Intestinal and vaginal mucosa, brain, kidney, gastric mucosa, pancreas, and salivary glands	Masopust et al., 2006, 2010; Hofmann and Pircher, 2011; Casey et al., 2012
	Vesicular stomatitis virus infection	Brain, salivary glands, and intestinal mucosa	Masopust et al., 2004; Klonowski et al., 2004; Wakim et al., 2010; Hofmann and Pircher, 2011
	Herpes simplex virus infection	Skin, dorsal root ganglia, and vaginal mucosa	Gebhardt et al., 2009, 2011; Tang and Rosenthal, 2010
	Vaccinia virus infection	Skin and intestinal mucosa	Isakov et al., 2009; Jiang et al., 2012
Non-specific local inflammation	DNFB treatment	Skin	Mackay et al., 2012a
	Nonoxynol-9 treatment	Vaginal mucosa	Mackay et al., 2012a
Local antigen presentation	Intracranial injection of peptide-pulsed DC	Brain	Wakim et al., 2010
Non-specific T cell activation	Lymphopenia-driven T cell activation	Intestinal and vaginal mucosa, brain, kidney, gastric mucosa, pancreas, and salivary glands	Casey et al., 2012

Abbreviations: i.c., intracranial; DNFB, 1-fluoro-2,4-dinitrobenzene; DC, dendritic cells.

### Animal models describing CD103^+^ T_RM_ cells

**Figure 1:**
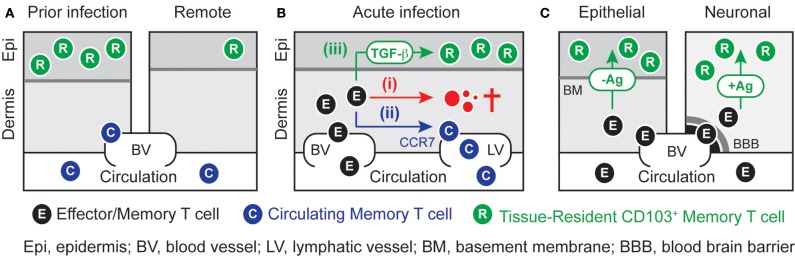
**Local differentiation and persistence of T_RM_ cells. (A)** Localized infection or inflammation of the skin generates a population of epidermal CD103^+^ T_RM_ cells that exist in disequilibrium with their counterparts in the circulation. High numbers of T_RM_ cells are concentrated at sites of previous infection or inflammation, whereas remote areas of skin contain only low numbers of these cells. **(B)** Effector CD8^+^ T cells that have infiltrated infected skin can undergo three principal fates. While the majority of cells are likely to (i) die *in situ*, others may (ii) egress the skin in a CCR7-depdendent fashion and join the evolving pool of circulating memory T cells. A minority of cells may (iii) access the epidermal layer of the skin where they can undergo TGF-β-dependent differentiation into T_RM_ cells during the resolution phase of infection. **(C)** The role of local antigen recognition in T_RM_-differentiation is dependent on anatomical location. While peripheral tissues such as skin and intestinal mucosa can promote T_RM_-differentiation in the absence of local antigenic stimulation, the same process in neuronal tissues such as brain and ganglia requires local antigenic T cell restimulation. The basement membrane in the skin and the blood-brain-barrier in the brain sequester T_RM_ cells from the circulation.

Skin infection with herpes simplex virus (HSV)-1 generates a population of CD8^+^ CD103^+^ T_RM_ cells in the epidermal layer of the skin that persist at elevated frequencies for more than a year after initial lodgment. Interestingly, these cells are concentrated at sites of previous infection or inflammation, while remote areas of skin harbor only low numbers of these cells (Figure 1A) (Gebhardt et al., 2009; Mackay et al., 2012a). Intravital imaging experiments have demonstrated that epidermal T_RM_ cells have an irregular shape with a dendritic morphology (Gebhardt et al., 2011) and thereby resemble other immune cell types present in the same location including DETC and Langerhans cells. Consistent with their site-specific accumulation and persistence, epidermal T_RM_ cells show only very restricted local migration and minimal displacement over time. Notably, this behavior is in stark contrast to the rapid movement seen for CD4^+^ memory T cells which, in contrast to CD8^+^ T cells, mainly localize to the dermal layer of the skin (Gebhardt et al., 2011).

Similar populations of persisting CD8^+^ T_RM_ cells have been described following virus infection of the brain and submandibular glands (Hawke et al., 1998; Wakim et al., 2010; Hofmann and Pircher, 2011), and intracranial labeling experiments have indicated that clusters of neuronal T_RM_ cells are retained in areas of previous infection for at least several weeks (Wakim et al., 2010). Such results are in line with the observation that intestinal infection generates a distinct population of virus-specific epithelial CD8^+^ memory T cells that differs from their counterparts in the lamina propria or the spleen in terms of T cell receptor rearrangement and usage (Isakov et al., 2009). Interestingly, T_RM_ cells isolated from the brain or gut show impaired survival and defective recall proliferative responses upon transfer into the circulation of recipient mice (Masopust et al., 2006; Isakov et al., 2009; Wakim et al., 2010). Thus, T_RM_ cells may irreversibly adapt to their local environments and therefore, may require a constant supply of microenvironment-specific factors for long-term survival and optimal functionality.

The most definitive pieces of evidence for the disequilibrium between T_RM_ and circulating memory T cells stem from experiments employing parabiotic mice (Klonowski et al., 2004; Jiang et al., 2012), tissue transplantation (Gebhardt et al., 2009; Masopust et al., 2010), and pharmacological blockade of T cell recirculation (Masopust et al., 2010; Jiang et al., 2012). In addition, experiments involving the transfer of male T cells into female recipients have demonstrated the long-term survival of epidermal T_RM_ cells even in the face of an anti-male histoincompatibility response that results in the rapid rejection of circulating T cells (Gebhardt et al., 2011). Collectively, these studies demonstrate that circulating memory CD8^+^ T cells have only very limited access to epithelial and neuronal compartments in the steady state. Instead, these anatomical niches are permanently occupied by an autonomous population of non-migratory T_RM_ cells that are characterized by the expression of a unique pattern of adhesion and migration molecules (Table 2).

**Table 2:** **Phenotypic differences between CD8^+^ memory T cell subsets**.

**Category**	**Marker**	**T_CM_**	**T_EM_**	**T_RM_**
Migration/adhesion	CD62L	++	−	−
	CCR7	++	±	−
	CD44	++	++	+++
	CD69	−	−	++
	CD103	−	−	+++
	E-cadherin	−	−	+
	VLA-1	±	±	++
	ESL^a^, PSL^a^	++/−	−	+++
Survival	CD122	++	+	+
	CD127	++	+	+
	Bcl-2	++	++	++
Effector function	Granzyme B	−	+	+

a Shown only for skin T_RM_ cells.

Abbreviations ESL E-selectin ligands PSL P-selectin ligands.

Non-migratory peripheral cells may also exist amongst the pool of CD4^+^ memory T cells (Cauley et al., 2002; Clark et al., 2006). For instance, a recent report has described a population of lung-seeking CD69^+^ CD11a^+^ memory CD4^+^ T cells that were retained in the lung parenchyma for at least 3 weeks in parabiotic mice (Teijaro et al., 2011), although others have reported a progressive decline of virus-specific CD4^+^ memory T cells in the same location (Cauley et al., 2002). A similar population of lung-resident CD4^+^ memory T cells with a distinct phenotype has also been described in human lungs (Purwar et al., 2011), and given that these cells were enriched for influenza virus-specific cells, it appears likely that these cells play an important role in early local protection from reinfection (Hogan et al., 2001b; Purwar et al., 2011). While in previously HSV-infected mouse skin almost all CD8^+^ memory T cells have hallmark features of T_RM_ cells, including epidermal localization and expression of high levels of CD103 (Gebhardt et al., 2011), the pool of peripheral CD4^+^ memory T cells in the skin and other peripheral organs may be considerably more heterogeneous. This is supported by the observation that virus-specific CD4^+^ memory T cells consist of both CD103^−^ and CD103^int^ populations in previously infected skin (Gebhardt et al., 2011). Some of these cells may represent truly recirculating cells in transit through the periphery, as indicated by lymph canulation studies (Mackay et al., 1990; Yawalkar et al., 2003), whereas others might be retained in extralymphoid locations for prolonged periods of time (Teijaro et al., 2011). Further studies will have to clarify whether such cells are indeed permanently resident within peripheral tissues and, if so, whether similar mechanisms are involved in the long-term persistence of both CD4^+^ and CD8^+^ T cells subsets.

### Human CD8^+^ CD103^+^ memory T cells with characteristics of T_RM_ cells

The expression of CD103 defines a heterogeneous population of intraepithelial T cells in skin and mucosal tissues that exist in many species including rodents and humans (Parker et al., 1992; Agace et al., 2000). Intraepithelial populations of T cells comprise non-conventional T cells expressing CD8α:α homodimers and/or γδ T cells, as well as conventional CD8α:β αβ T cells (Lefrancois et al., 1994; Hayday et al., 2001). While these cells are present in many tissues under normal physiological conditions, a marked accumulation of the CD8^+^ CD103^+^ αβ T cell subset is often associated with localized immune phenomena in epithelial and neuronal compartments, including graft rejection (Hadley et al., 1999; Wong et al., 2003), chronic inflammation (Ebert et al., 1998), infection (Piet et al., 2011), tumor development (Ling et al., 2007; Masson et al., 2007; Webb et al., 2010), and autoimmune processes (Pauls et al., 2001; Mizukawa et al., 2002; Teraki and Shiohara, 2002). Intriguingly, there is accumulating evidence that such populations of human T cells share major characteristics with the T_RM_ cells described in mouse experiments, including their long-term persistence and expression of T_RM_ signature markers such as CD69 and VLA-1, in addition to CD103.

Psoriasis is a chronic inflammatory skin disease characterized by well-demarcated erythematous plaques that tend to reoccur in identical anatomical locations on cessation of therapy (Nestle et al., 2009). Of note, psoriatic lesions are characterized by the accumulation of epidermal CD8^+^ CD103^+^ CD69^+^ memory T cells (Pauls et al., 2001; Teraki and Shiohara, 2002). Most strikingly, studies using xeno-transplantation of healed skin onto immuno-compromised mice have shown that these cells can locally persist for several weeks after transplantation, and that their activation by as yet unknown antigens results in the VLA-1-dependent local reappearance of psoriatic lesions (Boyman et al., 2004; Conrad et al., 2007). Another example for the contribution of resident T cells to localized skin pathology is the so-called fixed drug eruption, an allergic skin reaction with sharply demarcated lesions that are triggered by the ingestion of certain drugs. Interestingly, lesion development is characterized by the rapid activation of epidermal CD8^+^ CD103^+^ memory T cells (Mizukawa et al., 2002). Of note, these lesions strictly reoccur in identical anatomical regions even several years after the last exposure to the disease-causing drug, implicating long-term survival of locally resident memory cells (Mizukawa et al., 2008). Furthermore, predilection sites for lesions are often areas of skin that have previously been exposed to some form of tissue trauma or infection (Mizukawa and Shiohara, 2002), thereby resembling the requirements for optimal T_RM_ cell lodgment described in mouse experiments (Gebhardt et al., 2009; Mackay et al., 2012a). Other examples of T_RM_ cells in humans include influenza virus-specific CD8^+^ CD103^+^ T cells that persist in the alveolar epithelium of the lungs (Piet et al., 2011), as well as virus-specific CD8^+^ memory T cells that are preferentially retained in close proximity to the epidermis and peripheral nerves following HSV-2 infection in vaginal skin (Zhu et al., 2007, 2009). In addition, Epstein-Barr virus-specific CD8^+^ CD103^+^ T cells have been detected at high frequencies in the tonsils of persistently infected individuals (Hislop et al., 2005; Woodberry et al., 2005). Collectively, these studies strongly suggest that permanently tissue-resident T cells equivalent to the CD8^+^ CD103^+^ T_RM_ cells defined in mice, also exist in humans. Importantly, these cells appear to play a significant role in local infection control and various types of immuno-pathologies.

## Local differentiation and persistence of CD103^+^ T_RM_ cells

Several lines of evidence indicate that the local differentiation of T_RM_ cells occurs early during the resolution phase of infection. For instance, circulating effector cells rapidly lose the ability to infiltrate epithelial tissues and form T_RM_ cells (Masopust et al., 2010; Mackay et al., 2012a). Moreover, substantial populations of male-derived T_RM_ cells can lodge in female recipient mice even in the face of a potent anti-male rejection response that results in the rapid elimination of virtually all effector cells from the circulation (Gebhardt et al., 2011). These results further suggest that cells capable of differentiating into T_RM_ cells must exist amongst the effector cells that infiltrate peripheral tissues at the early stages of infection. Following pathogen clearance, these effector cells may undergo three fundamentally different fates (Figure 1B). While the majority of cells are likely to undergo apoptotic cell death *in situ*, others may enter afferent lymphatics in a CCR7-dependent manner and return to the circulation where they can join the evolving pool of circulating memory cells (Brown et al., 2010; Jennrich et al., 2012). A minority of cells however, may access epithelial compartments and further differentiate into long-lived T_RM_ cells. Given that T cell receptor-dependent activation can reduce the responsiveness of effector T cells toward CCR7 ligands (Debes et al., 2004; Schaeuble et al., 2011; Jennrich et al., 2012), it is possible that local antigen recognition, although not strictly required (Casey et al., 2012; Mackay et al., 2012a), supports effector T cell retention and further T_RM_ cell differentiation. While commitment to either of these fates could be a stochastic process, it is tempting to speculate that the ability to differentiate into either circulating memory or T_RM_ cells may be specific traits of distinct committed precursor cells. Future studies will have to establish the nature of T_RM_ precursor cells and will also have to better define the environmental cues that support their differentiation into T_RM_ cells. Such insights may help to determine optimal conditions for the generation of high densities of T_RM_ cells, critical for the development of T cell-based future vaccines, as discussed below.

### The role of antigen depends on anatomical location

The molecular mechanisms that underlie the generation and long-term persistence of T_RM_ cells remain largely unknown. However, recent studies have begun to shed some light on the basic requirements for the establishment of peripheral T_RM_-cell populations. Although T cells typically require antigenic activation in order to infiltrate peripheral sites, their subsequent differentiation into T_RM_ cells in extralymphoid tissues does not require ongoing stimulation (Figure 1C). Non-specific inflammation alone for instance is sufficient to generate a substantial population of T_RM_ cells that locally persist for more than a year (Mackay et al., 2012a). Remarkably, even lymphopenia-driven activation following transfer of transgenic T cells into lymphocyte-deficient mice results in the generation of T_RM_ populations in a broad variety of peripheral organs (Casey et al., 2012). Exceptions to this, however, are neuronal tissues such as the brain and dorsal root ganglia (DRG) where local recognition of cognate antigen is required for CD103 induction in CD8^+^ T cells (Figure 1C) (Wakim et al., 2010; Casey et al., 2012; Mackay et al., 2012a). Interestingly, even the intracranial injection of peptide-pulsed dendritic cells is sufficient to generate a local population of T_RM_ cells in the brain (Wakim et al., 2010). The additional level of “specificity control” in neuronal tissues may represent a mechanism to restrict the lodgment of potentially autoaggressive T cells, which could have detrimental consequences in the face of the inherently limited regenerative capacity of these organs. This further suggests that autocrine factors produced by the T cells themselves are involved in these specific locations, whereas other tissues such as the epidermis and the intestinal epithelium may promote T_RM_ differentiation through the provision of such factors in a more promiscuous and paracrine manner.

### The role of transforming growth factor-β

Transforming growth factor (TGF)-β induces CD103 expression in activated rodent and human T cells (Kilshaw and Murant, 1990; Cepek et al., 1993). Accordingly, epithelial T cells expressing a dominant negative form of the TGF-β receptor show impaired upregulation of CD103 *in vivo*. This has been demonstrated in several experimental models including intestinal graft-versus-host disease, pulmonary influenza virus infection and lymphopenia-driven induction of intestinal epithelial T cells (El-Asady et al., 2005; Lee et al., 2011; Casey et al., 2012). Thus, TGF-β-mediated signals play a critical role for CD103 induction during T_RM_ differentiation in various body locations (Figure 1B). Nevertheless, it remains possible that other local factors are necessary to drive and sustain the remarkably elevated levels of CD103 expression seen particularly for epithelial T cells such as T_RM_ cells and DETC. Several cell types including fibroblasts, mast cells, T cells as well as keratinocytes and enterocytes can be potent sources of TGF-β, particularly during the regenerative wound-healing phase of the immune response (Li et al., 2006). In addition, epithelial injury results in the upregulation of the integrin α_*v*_β_6_ (Breuss et al., 1995) that is involved in the local conversion of latent TGF-β into its biologically active form (Li et al., 2006). For instance, cutaneous wounding induces α_*v*_β_6_ expression selectively in keratinocytes surrounding the wound edges (Breuss et al., 1995), which suggests that these cells may be involved in providing and converting active TGF-β for the induction of CD103 in differentiating T_RM_ cells. The assumption that epithelial accumulation of CD103^+^ T cells is a consequence of local induction rather than selective recruitment of CD103^+^ cells is further supported by the observation that effector cells can readily access epithelial and neuronal sites in the absence of CD103 expression and that, in fact, the vast majority of early infiltrating cells lack expression of this integrin subunit (Wakim et al., 2010; Mackay et al., 2012a).

### The role of the α_E_β_7_ integrin

CD103, the α-chain of the α_E_β_7_ integrin, is one of the signature markers for CD8^+^ T_RM_ cells and functions as a receptor for E-cadherin, an adhesion molecule specifically expressed by epithelial cells (Cepek et al., 1993, 1994). While some effector CD8^+^ T cells express killer lectin receptor G1 (KLRG1), which is another E-cadherin binding molecule (Grundemann et al., 2006), the expression of this molecule is usually absent from T_RM_ cells (Masopust et al., 2006; Wakim et al., 2010; Hofmann and Pircher, 2011). Intestinal epithelial T cells and DETC can use CD103 to adhere and interact with enterocytes and keratinocytes, respectively (Cepek et al., 1993, 1994; Schlickum et al., 2008). In line with this, CD103-deficient mice have reduced numbers of these T cells (Schon et al., 1999, 2002; Schlickum et al., 2008), although residual populations are still present even in the absence of CD103. Furthermore, virus-, tumor-, or alloantigen-specific CD8^+^ T_RM_ cells deficient in CD103 expression show impaired persistence in the brain, lung and the intestinal epithelium (El-Asady et al., 2005; Masson et al., 2007; Wakim et al., 2010; Lee et al., 2011; Casey et al., 2012). These results implicate a functional role for CD103 in the local differentiation and long-term persistence of T_RM_ cells in various epithelial and neuronal tissues, although the requirement of CD103 may not be absolute (Lefrancois et al., 1999). Interestingly, T_RM_ cells themselves also express E-cadherin and this molecule is involved in optimal persistence of virus-specific T_RM_ cells in salivary glands (Hofmann and Pircher, 2011). While binding of CD103 to E-cadherin is likely to promote local retention by tethering T cells to neighboring epithelial cells, other modes of action apart from this adhesive function have also been proposed. These include the regulation of T cell proliferation and cytolytic effector function, but possibly also extend to the functional modulation of epithelial and T cells as a result of E-cadherin ligation by T cell-derived CD103 (Agace et al., 2000). Moreover, CD103^+^ T_RM_ cells in the brain express higher levels of the anti-apoptotic molecule Bcl-2 in comparison to CD103^−^ cells isolated from the same location, implicating a role for outside-in signaling through CD103 in promoting T_RM_ cell survival (Wakim et al., 2010). This mechanism may be of particular importance given that T_RM_ cells show only minimal homeostatic turnover when compared to lymphoid CD8^+^ memory T cells (Masopust et al., 2006; Gebhardt et al., 2009; Wakim et al., 2010), indicating that the maintenance of the T_RM_ pool is mainly regulated by their longevity rather than through constant self-renewal. Consistent with this, T_RM_ cells show only low-level expression of CD122 (Masopust et al., 2006; Gebhardt et al., 2009; Wakim et al., 2010), the receptor for interleukin (IL)-15 known to drive homeostatic turnover in lymphoid CD8^+^ memory T cells (Surh and Sprent, 2008), and it has been suggested that CD8^+^ memory T cells can be maintained independently of IL-15 signals following mucosal, as in contrast to systemic infections (Verbist et al., 2011).

### The putative role of other adhesion and migration molecules

Apart from CD103, T_RM_ cells also express a number of other adhesion and migration molecules that are likely to be involved in their local retention. These include the α_1_β_1_ integrin (VLA-1), CD44, ligands for E- and P-selectins, and the chemokine receptor CCR9, although expression of some of these molecules may be specific for T_RM_ populations in certain organs (Table 2). T_RM_ cells in the epidermis and the salivary glands express high levels of VLA-1 (Gebhardt et al., 2009; Hofmann and Pircher, 2011), a receptor for the extracellular matrix (ECM) proteins collagen type I and IV (Ben-Horin and Bank, 2004), the latter of which is a major structural component of basement membranes. Of note, VLA-1^+^ influenza virus-specific CD8^+^ memory T cells in the lung localize to collagen-rich areas in close association to airways, blood vessels and alveoli, and genetic VLA-1 deficiency or antibody-mediated blockade of VLA-1 function results in strongly reduced numbers of these cells (Ray et al., 2004). It is still under debate as to whether lung-resident CD8^+^ memory T cells share major characteristics such as longevity and permanent residency with their counterparts in other extralymphoid tissues (Takamura et al., 2010; Lee et al., 2011), and this matter is further complicated by inherent technical difficulties in identifying bona fide tissue-resident as in contrast to intravascular T cells in this particular organ (Anderson et al., 2012). Regardless, these results nevertheless suggest that T_RM_ cells in other organs may also utilize the VLA-1 integrin for their adhesion to basement membranes in epithelial compartments. In a similar fashion, CD44, which is expressed at very high levels on T_RM_ cells (Gebhardt, unpublished results), may also be involved in local retention through its interaction with hyaluronic acid, a major ECM component abundantly expressed in epithelial tissues (Baaten et al., 2012). Furthermore, epidermal T_RM_ cells express remarkably high levels of ligands for E- and P-selectins (Jiang et al., 2012), which represent a heterogeneous group of modified glycoproteins including CD44, CD43, PSGL-1, and possibly others (Woodland and Kohlmeier, 2009). While these molecules play a major role in skin migration of effector and memory T cells through their binding to E- and P-selectin on endothelial cells in the cutaneous microvasculature (Agace, 2006), skin T_RM_ cells may further utilize such receptors to bind to as yet unidentified ligands constitutively expressed in the epidermis. Finally, T_RM_ cells can also express organ-specific chemokine receptors that enable them to respond to gradients of locally produced chemokines. One example of this is the sustained expression of CCR9 by gut-resident T_RM_ cells (Masopust et al., 2010), which is the receptor for the chemokine CCL25, constitutively expressed by enterocytes in the small intestine (Agace, 2006). Interestingly, another adhesion molecule critically involved in gut migration by virus-specific effector CD8^+^ T cells, the integrin α_4_β_7_ that binds to the mucosal addressin MAdCAM-1 on blood vessels (Williams and Butcher, 1997; Lefrancois et al., 1999; Agace, 2006), is rapidly lost in intestinal T cells on epithelial entry (Masopust et al., 2010), and therefore, does not seem to be involved in local persistence of gut T_RM_ cells.

Future studies will have to clarify the precise contribution of the aforementioned adhesion and migration receptors to the local retention and persistence of T_RM_ cells in different anatomical locations. Experimental approaches targeting selected candidate molecules, however, could be complicated by the possibility that many of these molecules may exert redundant or overlapping functions. Furthermore, in addition to their function in tethering T_RM_ cells to their microenvironments, several of these molecules have also been implicated in outside-in signaling in T cells, meaning that their ligation by ECM components may directly impact on cellular functions including long-term survival or the expression of preformed effector molecules such as granzyme B (Agace et al., 2000; Richter and Topham, 2007; Baaten et al., 2012). In line with this, T_RM_ cell express high levels of the activation marker CD69 even in the absence of cognate antigen (Masopust et al., 2006; Gebhardt et al., 2009), possibly reflecting some level of cellular activation maintained by their prolonged interaction with various microenvironmental stimuli.

## Local immunity by T_RM_ cells

While circulating memory T cells provide efficient protection against systemic infections that result in rapid accumulation of pathogens in lymphoid filter organs, their ability to deal with localized infections in the periphery is often limited. This deficiency is partly explained by their progressive loss of peripheral migration as well as by their delayed accumulation at peripheral sites of infection due to the time-consuming nature of T cell recruitment from the circulation. Instead, it has been proposed that memory T cells already residing in peripheral tissues at the time of infection—either permanently as in the case of T_RM_ cells, or temporarily as proposed for CD4^+^ memory T cells—are key to rapid infection control in barrier tissues such as skin and mucosa (Woodland and Kohlmeier, 2009; Di Meglio et al., 2011; Kupper, 2012; Masopust and Picker, 2012).

Importantly, memory CD8^+^ T cells isolated from the intestinal mucosa exhibit enhanced effector function such as rapid interferon (IFN)-γ production and target cell lysis following restimulation *ex vivo* (Masopust et al., 2001). Similarly, T_RM_ cells isolated from the brain and skin can rapidly reacquire effector functions and kill peptide-pulsed target cells *in situ* and *ex vivo*, respectively (Hawke et al., 1998; Wakim et al., 2010; Jiang et al., 2012). Our own unpublished results further indicate that virus-specific T_RM_ cells produce IFN-γ within the first 24 h after epidermal or mucosal infection with HSV-1. Such results demonstrate that T_RM_ cells indeed display the full arsenal of effector functions required for immediate local immune control at sites of peripheral infection.

### Control of low-level persistent and latent virus infections

As discussed above, peripheral infections leave behind a population of T_RM_ cells concentrated at sites of previous pathogen encounter. The reason why these cells are formed particularly in epithelial and neuronal tissues sequestered away from the circulation is not entirely clear. It is tempting to speculate, however, that their main evolutionary purpose is to deal with residual, chronic or latent reservoirs of pathogens. Of note, circulating CD4^+^ and CD8^+^ memory T cells are largely excluded from epithelial and neuronal tissues under non-inflammatory conditions (Klonowski et al., 2004; Gebhardt et al., 2011). Thus, in the absence of locally persisting T_RM_ cells, such tissues would be highly vulnerable to pathogens that specifically target these sites to establish locally persisting or latent reservoirs in the absence of overt inflammation (Zinkernagel, 2002), as seen in infections with HSV, varicella zoster virus, and human papilloma virus.

Epithelial HSV infection in the skin or vaginal mucosa results in the establishment of a latent infection that is restricted to sensory ganglia innervating the site of primary infection. This latent infection is characterized by the absence of both infectious viral particles and ongoing inflammation (Stanberry, 1992; Sawtell, 1997) and therefore escapes immuno-surveillance by circulating CD8^+^ memory T cells (Himmelein et al., 2011). However, T_RM_ cells selectively retained within infected ganglia (Khanna et al., 2003) can recognize latently infected neurons and control viral latency through non-cytolytic mechanisms including the site-directed release of effector molecules such as granzyme B (Van Lint et al., 2005; Knickelbein et al., 2008; Mackay et al., 2012b). Interestingly, these ganglionic T_RM_ cells retain their full functionality even in the face of chronic antigenic stimulation and can readily produce effector cytokines and mount local proliferative recall responses on experimental virus reactivation (Wakim et al., 2008; Mackay et al., 2012b). Importantly, similar populations of HSV-specific CD8^+^ T cells are also retained in latently infected ganglia in humans (Verjans et al., 2007), and further accumulate at sites of anticipated virus reemergence such as the sensory nerve endings and epidermis of previously HSV-2-infected human vaginal skin (Zhu et al., 2007, 2009).

### Protection from virus reinfection

The local persistence of T_RM_ cells at sites of previous infection or inflammation can be interpreted as a strategy of the immune system to predict or anticipate the site of reencounter with the same or similar pathogens (Bevan, 2011). This is best documented for skin infection, where levels of site-specific immunity in previously infected areas of skin can be compared with control skin not involved in the primary infection. Indeed, following localized skin infection with HSV-1 in B cell-deficient mice, there is strongly enhanced local protection from virus reinfection specifically at the site of previous infection harboring elevated numbers of T_RM_ cells (Gebhardt et al., 2009). Furthermore, following reconstitution of lymphocyte-deficient mice with virus-specific CD8^+^ T cells, T_RM_ cells can mediate site-specific immunity alone, in the absence of virus-specific antibodies and CD4^+^ memory T cells (Gebhardt et al., 2009). Importantly, this form of local immunity is long-lived and can be observed for at least 3 months after primary immunization (Gebhardt et al., 2009), which is consistent with the remarkable longevity of T_RM_ cells. Confirming these results, T_RM_ cells generated in skin and salivary glands after vaccinia or LCMV infection, respectively, mediate potent protection from rechallenge infection even when T cell recirculation is pharmacologically inhibited by treatment with the sphingosine-1-phosphate antagonist FTY720 (Liu et al., 2009; Hofmann and Pircher, 2011; Jiang et al., 2012). Similarly, memory T cells in the lung can contribute to local immunity (Hogan et al., 2001a; Ray et al., 2004), although protection from heterosubtypic reinfection in this location appears to wane with time (Liang et al., 1994), which is potentially related to the fact the lung environment is not as conducive to support T_RM_ cell survival as other barrier tissues (Woodland and Kohlmeier, 2009). Collectively, these results strongly support the notion that T_RM_ cells at body surfaces play a major role in protection from localized reinfection, whereas circulating CD8^+^ memory T cells may be specialized in dealing with pathogens that access lymphoid tissues following systemic infection.

### Protection from *de novo* infection—T_RM_ cells as vaccine targets

The induction of long-lived memory T cells able to patrol through peripheral tissues has proven difficult in the absence of continued T cell activation (Kundig et al., 1996; Bachmann et al., 1997; Hansen et al., 2009). However, T_RM_ cells do not rely on persisting antigen (Casey et al., 2012; Mackay et al., 2012a), and as a consequence, targeting these cells with novel vaccination strategies has the potential to overcome obstacles originating from the progressive loss of peripheral immune surveillance by circulating CD8^+^ memory T cells. Given that T_RM_ cells can mediate rapid immune control at peripheral sites of infection, the generation of high densities of such cells is an appealing goal for future vaccines against pathogens that invade the body via peripheral portals of entry.

**Figure 2:**
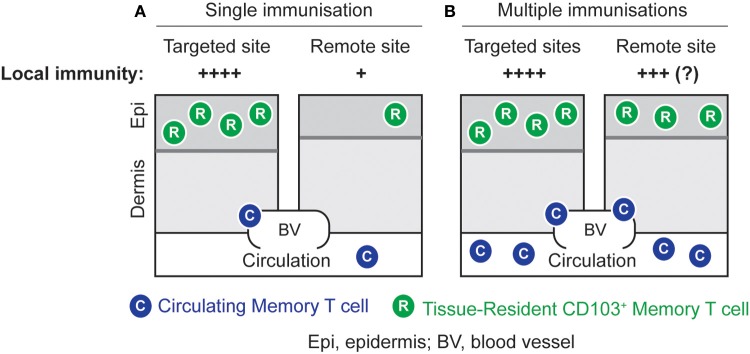
**Local immunity by site-specific or global lodgment of T_RM_ cells. (A)** High numbers of T_RM_ cells can be generated in the skin by an immunization strategy that combines the induction of effector T cells with the local application of an inflammatory stimulus. This results in potent local immunity at the targeted site. **(B)** Repeated immunizations in different skin sites result in elevated numbers of T_RM_ cells in both targeted and remote areas of skin. This strategy has the potential to generate global T_RM_-cell-mediated immunity against skin infection, although this remains to be formally proven.

Strategically well-positioned at peripheral sites, T_RM_ cells may provide a first line of defense potent enough to control peripheral infection before local pathogen replication results in the establishment of chronic or latent reservoirs as seen in infections such as HSV and human immunodeficiency virus. We have recently shown that the combination of T cell activation and site-specific lodgment by non-specific inflammation generates high densities of long-lived T_RM_ cells in the skin and these that cells are able to (1) control local virus replication, (2) prevent viral skin disease, and (3) significantly reduce the ensuing latent infection in DRG innervating the site of *de novo* challenge infection (Mackay et al., 2012a). While this approach provides regional rather than global protection of the skin, it has recently been suggested that repeated immunization in prime-boost settings can generate high densities of T_RM_ cells even in remote areas of skin not involved in successive immunization steps (Jiang et al., 2012) (Figure 2). Furthermore, non-specific inflammation of the vaginal mucosa has been shown to result in the local accumulation of T_RM_ cells capable of controlling a subsequent infection with HSV-1 (Mackay et al., 2012a). Of note, the latter approach represents an example where coverage of a whole organ system, the cervico-vaginal mucosa, is achievable by a single intervention without the need of repeated immunizations. Collectively, these results provide promising proof-of-principle evidence that T_RM_ cells may successfully be exploited in vaccination settings. Future studies are needed to refine the requirements for optimal T_RM_ lodgment and coverage of different organ systems. Finally, complementing such innovative immunization strategies with the simultaneous induction of other adaptive immune elements, including tissue-tropic CD4^+^ memory T cells and antibody-secreting plasma cells, has the potential to further enhance protection afforded by T_RM_ cells.

## Concluding remarks

To a large extent, the field of T cell memory has been dominated by the paradigm that memory T cells are constantly recirculating cells that patrol through either lymphoid or extralymphoid tissues as a consequence of subset-specific migratory programs. An important extension to this concept is that these fundamentally different migration patterns correlate with the functional status exhibited by distinct memory subsets, and thereby, predict their participation in different types of immune responses. Recent evidence based on the analysis of antigen-specific memory T cells in various lymphoid and non-lymphoid tissues, however, suggests that a large proportion of peripheral CD8^+^ memory T cells are permanently sessile and non-migratory and therefore, do not easily fit into the prevailing concept of circulatory immune surveillance. Given that these T_RM_ cells show remarkable similarities in terms of the expression of core signature markers, residency and effector function irrespective of their mode of generation and localization to different peripheral organs, it is tempting to speculate that T_RM_ differentiation represents a default pathway of CD8^+^ memory T cell generation in non-lymphoid tissues. Their important role in infection control highlights the need to better understand the molecular mechanisms that govern their differentiation and long-term persistence in peripheral sites. Future studies will also have to clarify whether similar populations of non-migratory peripheral cells also exist among the pool of CD4^+^ memory T cells. Refining the contribution of circulating versus permanently tissue-resident populations of memory T cells to immune protection in barrier tissues such as the skin and mucosa will be critical to the design of future immunization strategies harnessing cellular immunity to protect against pathogens that invade through peripheral portals of entry.

### Conflict of interest statement

The authors declare that the research was conducted in the absence of any commercial or financial relationships that could be construed as a potential conflict of interest.
